# The Role of Acoustic Distance and Sociolinguistic Knowledge in Dialect Identification

**DOI:** 10.3389/fpsyg.2018.00818

**Published:** 2018-07-18

**Authors:** Hanna Ruch

**Affiliations:** University Research Priority Programme Language and Space, University of Zurich, Zurich, Switzerland

**Keywords:** dialect identification, salience, acoustic distance, sociolinguistic knowledge, Swiss German, Grison German, Zurich German

## Abstract

Listeners are able to quite accurately distinguish between different dialects of their native language, but little is known about the process of dialect identification and the phonetic cues listeners use to identify someone’s regional origin. This study examines how different segments, acoustic between-dialect distance, and the listeners’ knowledge about a dialect contribute to this process. Native speakers of Grison and Zurich German were asked to categorise isolated words spoken by eight speakers of Grison and eight speakers of Zurich German. Stimuli contained either none, one, or two segmental cues to regional origin. The presence of one dialect-specific segment was enough to allow for an identification rate well above chance. Sensitivity measures and analysis of reaction time showed that the two dialect groups largely relied on the same segmental cues. Acoustic distance to the other dialect, quantified as Euclidean distance in the F1 × F2 vowel space, generally facilitated dialect identification, but interacted with native speakers’ knowledge about the dialects: in segments which listeners explicitly associated with one of the two dialects, acoustic distance facilitated dialect recognition to a larger extent than in segments in which listeners were not aware of dialectal variation. The results suggest that, depending on the listener’s prior knowledge about a dialect, acoustic variation is weighted differently. Further analysis showed that Zurich listeners were more sensitive to the dialect differences, responded faster, and presented a more marked own-dialect response bias than Grison listeners. These findings are in line with the status of Grison German as a marked dialect and Zurich German as a neutral dialect, and suggest that, depending on their own dialect’s status, listeners used different decision strategies.

## Introduction

When listening to someone speaking, we quite quickly infer from their speech whether they are a native speaker of that language, whether they speak the same dialect as we do, and if not, where they might come from. How fast and how accurately listeners recognise different dialects is relevant for sociolinguistics and social psychology because listeners use accents and dialects in social categorization (e.g., Rakić et al., 2011). Therefore, whether or not a specific accent is recognised affects the kind of stereotypes evoked (see Van Bezooijen and Gooskens, 1999), and recent research has provided evidence that a specific regional accent can affect friendship and cooperative preferences (e.g., Cohen and Haun, 2013; Heblich et al., 2015). Dialect identification is also of interest to psycholinguistics, given that listeners have been shown to perceptually adapt to phonetic variability (Kraljic et al., 2008), including regional accents (Trude and Brown-Schmidt, 2012). In some cases, the activation of a specific social category using verbal labels or symbolic elements was enough to shift the listeners’ phonetic categories (Niedzielski, 1999; Hay and Drager, 2010). It is conceivable that stereotyped linguistic forms (shibbolets) may have a similar effect on speech processing and comprehension by activating a specific language variety in the listener.

### Dialect Recognition

Research on different languages has shown that listeners are able to localise, with more or less precision, a specific regional accent (for overviews, see Clopper and Pisoni, 2005; McKenzie, 2015). Most studies played longer samples of spontaneous or read speech, of about 5 and up to 45 s, to the listeners. A variety of dialect identification tasks were used depending on the aim of the research, for instance, free classification (Clopper and Pisoni, 2007; Bent et al., 2016; Jones et al., 2017), ladder task (e.g., Bent et al., 2016), forced-choice localizations at different levels of geographic specificity (Van Bezooijen and Gooskens, 1999), or forced-choice identification of dialects using common denominations (Guntern, 2011; Leemann et al., 2016). Independently of the method used, previous research suggests that listeners are able to identify a regional variety above chance level. Accuracy is difficult to compare across studies due to differences in experimental design such as stimulus length, number of response options, or the specific task. However, several more general, listener-related factors seem to positively affect accuracy in dialect identification. Not surprisingly, previous experience with the tested dialects, for instance, through moving or traveling, facilitates their recognition (Clopper and Pisoni, 2004a; Díaz Campos and Navarro Galisteo, 2009). The finding that listeners more easily identified their own over other dialects (Williams et al., 1999; Gooskens, 2005; Boomershine, 2006; Baker et al., 2009; Yan, 2015; Avanzi and Boula de Mareüil, 2017) has also been explained with exposure and experience (Clopper and Pisoni, 2004a; Baker et al., 2009; Yan, 2015). An alternative explanation, however, is that an own-dialect bias accounts for the higher accuracy (see Ruch, in press), similar to an own-age bias in the recognition of speaker age (Moyse et al., 2014). The present paper will specifically address this question. Apart from a potential own-dialect bias, listeners have been found to ascribe ambiguous stimuli to the most prestigious variety that was offered as an answer. For instance, in experiments on American English varieties (Purnell et al., 1999; Perrachione et al., 2010), Mandarin dialects (Yan, 2015), and French regional accents (Avanzi and Boula de Mareüil, 2017), listeners were biased towards ascribing most stimuli to Caucasian American English, Enshi Mandarin, and Parisian French, respectively. Furthermore, regional varieties that are present in the media seem to be identified more easily, especially when they appear in a clearly identifiable regional context, for instance, in the speech of well-known public persons (Lameli et al., 2008; Purschke, 2012).

From direct comparisons using acoustically manipulated stimuli (Van Bezooijen and Gooskens, 1999; Gooskens, 2005; Leemann and Siebenhaar, 2008; Fuchs, 2015; Leemann et al., 2016), it seems clear that listeners primarily rely on segmental cues, with prosodic cues playing a minor role. However, which segmental cues most directly contribute to dialect identification in a specific language is less clear, given that most studies have used longer speech samples which contain several potential cues to regional origin. One way of exploring the role of different segmental cues in dialect identification is *post hoc* acoustic analysis of the stimulus materials. Clopper and Pisoni (2004b) found that for American English dialects, several acoustic measures, in particular, vowel quality, significantly predicted to which region a stimulus was assigned. Only a few studies have investigated the role of acoustic differences in isolated words. Fridland et al. (2004) manipulated vowel quality in monosyllabic words to test which of the manipulated vowels facilitated recognition that the speaker was perceived as having a Southern American English accent. It was found that diphthongs, which are usually longer than monophthongs, as well as phonetic variants that occur in a small geographic area, were more often correctly categorised (see Graff et al., 1986, for a similar study on ethnic accent categorization). Boomershine (2006) and MacLeod (2012) used unmanipulated words and non-words, respectively, of which each included one segmental cue to regional origin. Both studies found that the specific segments that occurred in the word affected how well listeners identified the speaker’s dialect. From these results, it appears that features speakers overtly comment on [stereotypes in Labov’s (1972) terms] are the ones that contribute most to dialect recognition.

From previous research it thus seems conceivable that listeners identify dialects based on both acoustic detail as well as explicit knowledge such as dialect stereotypes. However, little is known about how these two types of information interact in dialect recognition. A first aim of this study is to quantify the contribution of several segmental dialect features to dialect identification. Their role will be tested using natural, unmanipulated speech materials in a forced-choice dialect identification task. Isolated words will be used which contain either none, one, or two segmental cues to the regional origin of a speaker. With the present approach it will be possible to isolate and order the effect of different segments to dialect recognition without having to distort the acoustic signal. Testing the effect of different segments to dialect identification is a way to quantify their perceptual salience (MacLeod, 2012, 2015). Perceptual salience can be defined as the cognitive conspicuousness of a (linguistic) feature (Lenz, 2010) and is considered to affect different linguistic processes, for instance, sound change (Ohala and Kawasaki, 1984) and phonetic accommodation (Trudgill, 1986; Babel, 2010; Walker and Campbell-Kibler, 2015). A symmetric design will be used in which speakers of two Swiss German dialects, Grison and Zurich German, are asked to categorise stimuli from these two dialects. In addition to identification scores, reaction time (RT) will be analysed to assess the listeners’ confidence in their decisions. A second aim is to study the process of dialect recognition in more detail by focusing on two issues: the role of prior knowledge about the dialects, and the significance of acoustic distance between them. We will investigate how acoustic properties of the stimuli interact with the speakers’ knowledge about dialect features and expectations about the dialects. A third aim of the present study is to explicitly address the question of an own-dialect bias. By using signal detection theory the listeners’ sensitivity to the dialect differences can be studied separately from a potential response bias. The findings will be interpreted in light of the dialect landscape and the particular sociolinguistic situation of German-speaking Switzerland, which will be introduced in the next subsection.

### Sociolinguistic Situation of German-Speaking Switzerland

Despite some tendencies of dialect leveling (Christen, 1997; Eckhardt, 2016), German-speaking Switzerland presents considerable dialectal variation in a relatively small geographic area (Christen et al., 2010; Glaser and Bart, 2015). Its sociolinguistic situation can be characterised as a medium-dependent diglossia (*mediale Diglossie*; Ammon, 1995) with dialect being spoken in all everyday situations, and standard German being the preferred variety for written communication. In inter-dialectal settings, instead of a regionally-accented standard variety, everybody speaks their own dialect, leading to a high number of dialect contact situations (Christen, 1997). Furthermore, spoken dialects are often used in the media, so that Swiss Germans are exposed to different dialects on a daily basis and can be considered to possess broad knowledge about dialectal variation. Despite none of the dialects having the status of a standard variety, they differ significantly in terms of popularity (Werlen, 1985; Ris, 1992) and in reactions they evoke in listeners (Werlen, 1985; Leemann et al., 2015a,b). This situation, together with the lack of a prestigious spoken standard variety, provides an interesting test case for analysing the effects of sociolinguistic knowledge and acoustic details on dialect recognition.

The speakers of the present study come from two non-adjacent dialect regions: the Alpine Chur Rhine valley, and the region around Zurich, Switzerland’s biggest city. We will refer to the dialect spoken in the Chur Rhine valley as Grison German (GRG), and to the dialect spoken in and around Zurich as Zurich German (ZHG).

### Grison and Zurich German

Grison (GRG) and Zurich German (ZHG) are High Alemannic dialects and differ at all linguistic levels. According to dialect descriptions (Hotzenköcherle et al., 1962; Weber and Dieth, 1987; Eckhardt, 1991, 2016; Fleischer and Schmid, 2006; Christen et al., 2010), the main segmental differences between GRG and ZHG include the vowel system (especially the quality of the front vowels and word-final schwa), the realisation of word-initial and post-vocalic *k* as either [k^h^]/[kk] or a velar fricative/affricate, and the presence/absence of open syllable lengthening and nasal/liquid gemination. **Table 1** provides an overview of the main segmental differences between the two dialects. Throughout this paper, they will be referred to as (segmental) dialect features and will be represented by the capital letters in the leftmost column in **Table 1**. Our own phonetic analysis of 16 younger speakers from each of the two dialects confirmed the presence of the dialect features in **Table 1** and showed considerable synchronic variation for GRG /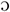/ and ZHG /æ/ (Ruch, 2015). GRG and ZHG also differ in a number of prosodic parameters as described by Leemann (2012). He found GRG intonation contours to show a high pitch phrase-initially, a more marked declination, and to be less influenced by lexical stress than ZHG intonation. Analysis based on crowded-sourced data (Leemann, 2016) further suggests that GRG is characterised by a slightly slower articulation rate in comparison to ZHG, confirming an earlier study (Leemann, 2012).

**Table 1:** Main segmental differences between Grison and Zurich German according to Hotzenköcherle et al. (1962), Fleischer and Schmid (2006), Ruch (2015), and Eckhardt (2016).

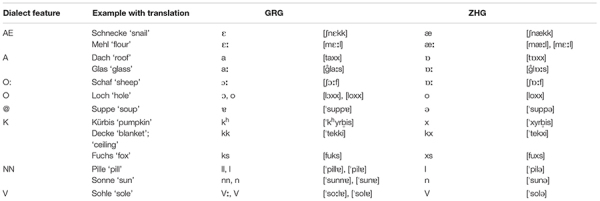

GRG and ZHG also differ in terms of social values ascribed to them. Listeners of different dialects described ZHG as being *ausgeglichen* ‘balanced,’ *gewöhnlich* ‘common,’ *eintönig* ‘monotonous,’ and *unsympathisch* ‘dislikeable.’ GRG was assigned adjectives such as *urchig* ‘down-to-earth,’ *originell* ‘original,’ *warm* ‘warm,’ and *schön* ‘beautiful’ (research reviewed in Werlen, 1985). By using audio stimuli to better separate judgments of the dialects from stereotypes attached to a region and its people (see Ris, 1992), Werlen (1985) found that ZHG was less negatively evaluated (see also Leemann et al., 2015a). Overall, the attitudes towards ZHG and GRG are clearly asymmetric (Ris, 1992) and seem to be shared among people from different regions (Werlen, 1985).

## Materials and Methods

### Stimulus Materials

The stimulus materials are listed in **Table 2** and included 48 different lexical items containing either none (12 items), one (22 items), or two segmental dialect features (14 items). The 12 stimuli without a segmental dialect feature, but containing potential prosodic differences, will serve as a baseline. For instance, apart from the documented segmental dialect features, the stimuli could further differ in prosodic aspects such as articulation rate, intonation, or the realisation of lexical stress (see section “Grison and Zurich German”). The 48 lexical items were spoken by eight female speakers from the Chur Rhine valley in the Grisons and eight female speakers from the region of Zurich. All speakers were between 19 and 24 years old and grew up in monolingual Swiss German homes in the respective region.

**Table 2:** Words (here written in Standard German) included for each segmental dialect feature and each condition.

	Dialect feature(s)	Stimuli
One segmental dialect feature	AE	Mehl ‘flour,’ Schnecke ‘snail’
	A	Mann ‘man,’ Glas ‘glass,’ Gras ‘grass,’ Hammer ‘hammer’
	O:	Strasse ‘street,’ Schaf ‘sheep,’ Haare ‘hair,’ Maler ‘painter’
	O	Loch ‘hole,’ Schloss ‘castle,’ Frosch ‘frog’
	@	Suppe ‘soup,’ Lupe ‘magnifier,’ Lunge ‘lung’
	K	Kürbis ‘pumpkin,’ Zucker ‘sugar,’ Decke ‘blanket,’ Wecker ‘alarm clock,’ Fuchs ‘fox,’ Luchs ‘lynx’
Two segmental dialect features	K, AE	Keller ‘cellar,’ Gepäck ‘luggage,’ sechs ‘six’
	K, A	Katze ‘cat,’ Dachs ‘badger,’ Lachs ‘salmon’
	K, O	Koch ‘cook,’ Block ‘notepad’
	NN, @	Sonne ‘sun,’ Spinne ‘spider,’ Brunnen ‘fountain’
	O:, @	schlafen ‘to sleep’
	A, @	Lampe ‘lamp’
	O, @	Flosse ‘fin’
No segmental dialect feature	e:	Tee ‘tea,’ See ‘lake,’ CD ‘cd,’ Himbeere ‘raspberry’
	o:	Ohr ‘ear,’ rot ‘red,’ Boot ‘boat,’ Moos ‘moss’
	u:	Maus ‘mouse,’ Schaum ‘foam,’ Tausend ‘thousand,’ Maurer ‘brick layer’

The 48 (lexical items) × 16 (speakers) = 768 audio files were cut at the manually-set word boundaries, and amplitude was equalised in Praat. In order to reduce the number of stimuli per listener, the 768 stimuli were rotated over participants in four different experiment versions. Each version comprised four tokens of each of the 48 lexical items: two tokens from two different ZHG speakers, and two from two different GRG speakers, resulting in 4 (tokens) × 48 lexical items = 192 stimuli per listener. In each experiment version, a total of 12 stimuli were presented from each speaker. Each participant was randomly assigned to one of the four experiment versions.

### Participants

Twenty three native speakers of ZHG (15 females and 8 males), and 21 native speakers of GRG (13 females and 8 males) participated in the experiment. Listeners were recruited among the students of the University of Zurich, a professional school in Zurich, and the college of higher education in Chur. The listeners of both dialect groups were enrolled in different study programmes ranging from biology to teacher-training, English language studies, physiotherapy, and law. At the time of the experiment, 8 out of the 21 Grison participants were living in Zurich. Participants were between 19 and 30 years old (mean age GRG listeners: 23.2, mean age ZHG listeners: 22.0 years). They gave written informed consent and were paid for their participation.

### Procedure

The procedure included two dialect identification tasks and a post-task questionnaire and lasted between 30 and 40 min. The first task was a forced-choice dialect recognition task in which the participants were asked to listen to a stimulus and categorise the dialect as either “Grison German” or “Zurich German” by pressing on one of two buttons as quickly as they could. This procedure was repeated for all 192 stimuli. In the second task, the participants listened to the same 192 stimuli, but this time they categorised the stimuli on a visual analogue scale as more (or less) typical for Grison or Zurich German, respectively. Finally, in an online questionnaire they were asked about the typical features of each of the two dialects, their exposure to Grison and Zurich German, and several questions related to the experiment. In this paper, we only report on the first dialect recognition task and the post-task questionnaire.

Both dialect identification tasks were implemented in PsychoPy version 1.82 (Peirce, 2007, 2009). Participants wore closed headphones of the type Beyerdynamic DT 770 PR. Experiments were run in a quiet room at one of the institutions mentioned above by one of three research assistants, and participants read the instructions to the tasks from the computer screen. Each trial in the forced-choice dialect categorization task started with a grey screen. After one second, the answer options appeared on the screen, and 500 ms later, the audio stimulus was played back to the listener. As soon as the subject pressed the button, a new trial started, again with a grey screen. In order to accustom participants to the task, a training block with six words (three per dialect in a randomised order) was included at the very beginning. These lexical items were spoken by different speakers than the 16 mentioned above.

The test materials were randomised in 12 blocks of 16 stimuli each in order to avoid that several stimuli from the same speaker or the same word might appear in a row. Each block contained only one stimulus from the same speaker. Dialect identification could potentially be facilitated by speaker identification, especially when an easily identifiable token from the same speaker was already heard before. To control for this eventual effect, the type of stimulus (with/without dialect features), as well as the different segmental dialect features, were balanced across blocks. The placement of the answer options (left/right) were equally distributed among the four experiment versions and remained stable within an experiment.

## Results

The results will be presented in three parts. Section “Effect of Segmental Dialect Feature on Dialect Identification” describes how a listener’s dialect, and how the specific segment in the stimulus, affects dialect identification. Additional analyses are conducted to ensure that performance did not change over the course of the experiment. Section “Role of Acoustic Distance” explores how the acoustic distance between the dialects affects their identification. Lastly, section “Knowledge of Dialect Features,” explores the extent to which the findings are consistent with the knowledge speakers of GRG and ZHG have about the two dialects in question.

### Effect of Segmental Dialect Feature on Dialect Identification

**FIGURE 1:**
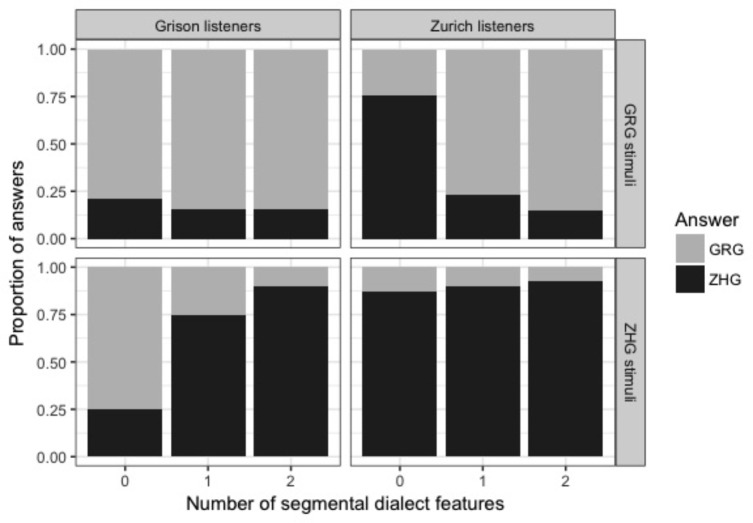
Distribution of dialect answers (black: ZHG; grey: GRG) according to a listener’s origin (columns; Grison and Zurich listeners) and the speaker’s dialect (rows; GRG and ZHG stimuli).

**Figure 1** shows the distribution of responses separately for Grison and Zurich listeners, speaker origin, and number of dialect features in the stimulus. Overall, participants were clearly above chance when categorising stimuli containing one or two segmental dialect features, and Zurich listeners showed slightly higher proportions of correct answers than Grison listeners (see **Table 3**). In stimuli without a segmental dialect feature, participants of both dialects were only slightly above chance and tended to attribute them to their own dialect, as is apparent from the high rate of correctly identified stimuli of the own, but not of the other dialect. In the subsequent analyses, signal detection theory will be applied to examine this bias, and to normalise for it when other aspects are in the focus of interest.

**Table 3:** Percentage of correct answers per listener group and condition.

Stimuli type	Grison listeners	Zurich listeners
No segmental dialect feature	51.9%	55.7%
One segmental dialect features	79.6%	83.3%
Two segmental dialect features	87.0%	88.8%
Overall	74.8%	78.0%

**FIGURE 2:**
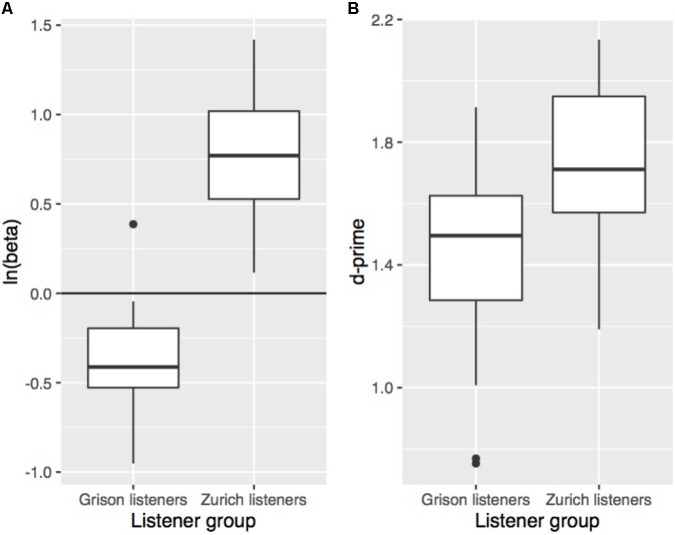
Response bias **(A)** and sensitivity **(B)** according to a listener’s origin (left: Grison listeners; right: Zurich listeners). The measures were calculated across conditions including all 48 words. Each boxplot contains one value per subject of the respective measure.

Signal detection theory is a method to calculate sensitivity (d′; i.e., the ability to distinguish between two stimuli) independently from response bias (Stanislaw and Todorov, 1999). This is done by correcting a participant’s hit rate (i.e., ZHG stimuli correctly identified as “Zurich German”) for their rate of false alarms (i.e., GRG stimuli erroneously identified as “Zurich German”). We calculated d′ using the loglinear approach to cope with hit and false alarm rates of 1 or 0 (Stanislaw and Todorov, 1999). A higher d′ value indicates a subject could more readily distinguish between the two dialects. To confirm the presence of a response bias as observed in **Figure 1** and to explore differences in response behaviour between the two listener groups, d′ and the natural logarithm of beta [i.e., ln(beta), a measure for response bias] were calculated by collapsing the data across conditions. Negative values of ln(beta) are indicative of a bias towards responding “Grison German,” and positive values signify a bias towards responding “Zurich German.” As evident from **Figure 2A**, both listener groups have a bias towards their own dialect, but this own-dialect bias is stronger for Zurich listeners. An unpaired *t*-test showed a significant effect of a listener’s dialect (*t*[42] = 4.0, *p* < 0.001) on the absolute value of ln(beta). Differences in sensitivity in the collapsed data are shown in **Figure 2B** and suggest that overall, Zurich listeners more readily distinguished between the two dialects. An unpaired *t*-test confirmed that d′ was higher for Zurich than for Grison listeners (*t*[42] = 3.5, *p* < 0.01).

As mentioned in section “Procedure,” listeners could potentially learn to identify a speaker by her voice, which would then lead to a better performance later in the experiment. If present, such an effect should be visible particularly in stimuli that are difficult to identify, that is, stimuli without segmental dialect features. To explore whether performance in difficult words increased over the course of the experiment, d′ was calculated for each of the 12 blocks separately for listener group and number of dialect features. Neither group showed an increase in d′ over the 12 blocks.

**FIGURE 3:**
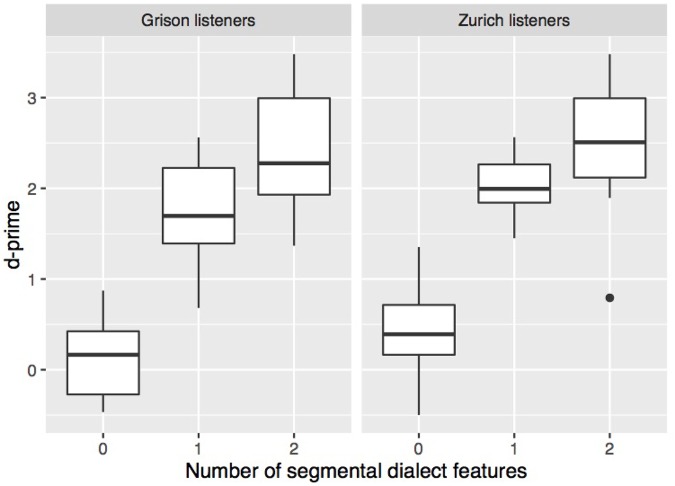
Sensitivity according to a listener’s origin and the experimental condition, i.e., stimuli containing none, stimuli with one, or with two segmental dialect features. Each boxplot contains for each listener one value of this measure.

For the subsequent analyses, d′ was calculated for each level of the independent variable in question. **Figure 3** shows that sensitivity increased with the number of segmental dialect features in the stimulus. A repeated-measures ANOVA with a listener’s dialect as a between-subjects factor and number of dialect features as a within-subjects factor showed a significant effect of number of dialect features (*F*[2,84] = 287.7, *p* < 0.001), but not of a listener’s dialect (*F*[1,42] = 4.0, *p* = 0.05). Pairwise *t*-tests with Bonferroni correction confirmed that the three stimulus types significantly differed from each other in both listener groups (Zurich listeners: *p* < 0.001 for comparisons 0–1 and 0–2; *p* < 0.01 for 1–2; Grison listeners: *p* < 0.001 for all pairwise comparisons). The presence of one or two segmental dialect features thus significantly improved the listeners’ ability to distinguish between the two dialects. Pairwise *t*-tests further showed that Grison and Zurich listeners differed in stimuli without and with one dialect feature with Zurich listeners being slightly more sensitive (*p* < 0.05). The results show that, despite potential prosodic cues in the stimuli, testing the effect of segmental cues using natural stimuli is possible, and they confirm the prevalence of segmental over prosodic cues observed in previous work.

**FIGURE 4:**
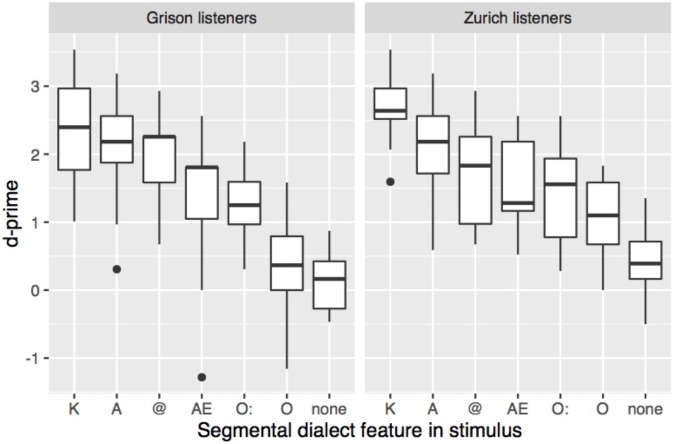
Sensitivity according to the segmental dialect feature in the stimulus. Stimuli with two segmental dialect features have been discarded from this analysis.

To explore the effect of the specific segments on dialect recognition, d′ was calculated separately for each listener and each dialect feature. The 14 stimuli with two dialect features (see **Table 2**) were excluded from the analysis because it remains unclear which of the two dialect features (if not both) facilitated dialect recognition (e.g., in *Katze* ‘cat,’ GRG [k^h^ats], ZHG [xts], a high d′ could be due to A, to word-initial K, or both). **Figure 4** shows how sensitivity varies with the segmental dialect feature in the stimulus. Stimuli with K, A, or schwa were more easily attributed to the correct dialect than stimuli with O:, O, or the baseline stimuli. A repeated-measures ANOVA showed a significant effect of dialect feature (*F*[6,252] = 76.6, *p* < 0.001), a listener’s dialect (*F*[1,42] = 5.5, *p* < 0.05), and an interaction between the two factors (*F*[6,252] = 2.9, *p* < 0.01). As shown in **Figure 4**, Grison and Zurich participants mostly relied on the same segmental features. Pairwise *t*-tests with Bonferroni correction indicated that among Zurich listeners, sensitivity was highest in stimuli with K, and lowest in stimuli without segmental dialect features. Among Grison listeners, K patterned with A and @, and O with the stimuli without dialect features, contributing the least to dialect recognition.

**FIGURE 5:**
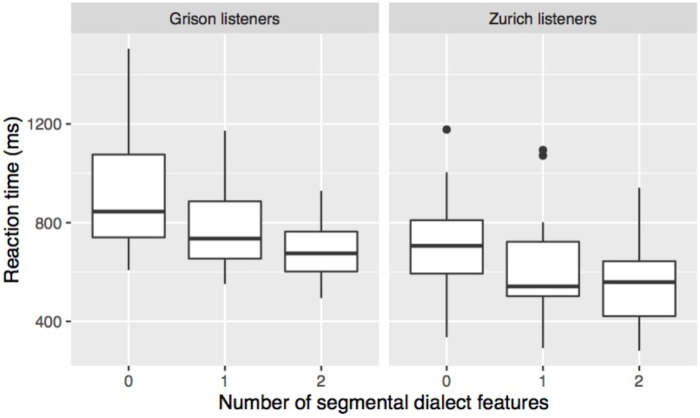
Reaction time (RT) according to a listener’s origin and the number of segmental dialect features in the stimulus. The boxplots contain one mean value of this measure for each subject and each condition.

RT was measured from the stimulus onset, and RT analyses were performed on the correct answers only. Tokens with very short (<500 ms from stimulus onset; 1 token) or very long response latency (>3 s from stimulus offset; 99 tokens; 1.5% of correct answers) were considered as outliers and removed from the analysis. As is typical for RT, the data distribution was right-skewed. The reciprocal Box-Cox-transformation was used because it most closely approximated normality. **Figure 5** shows that RTs were longer the fewer segmental dialect features occurred in a stimulus, and that Zurich listeners overall responded faster than Grison listeners. Linear mixed-effects models using the lme4 package in R (Bates et al., 2015) were performed on the transformed RTs with a listener’s dialect, number of dialect features, and stimulus duration as fixed factors. Stimulus duration was included in the model to control for differences in stimulus duration between words and speakers. The model with the best fit included random intercepts for speaker, word, and listener, and by-listener random slopes for number of dialect features. The inclusion of the random intercepts and slope was justified as shown by likelihood ratio tests (see Baayen et al., 2008). The interaction between a listener’s dialect and the number of dialect features was not significant (χ^2^[2] = 1.9, *p* = 0.40) so that it was removed from the model. The updated model showed a significant effect of a listener’s dialect (χ^2^[1] = 12.5, *p* < 0.001) and number of dialect features (χ^2^[2] = 12.7, *p* < 0.01), confirming that Zurich listeners responded faster and that the presence of one or two dialect features led to faster responses.

**FIGURE 6:**
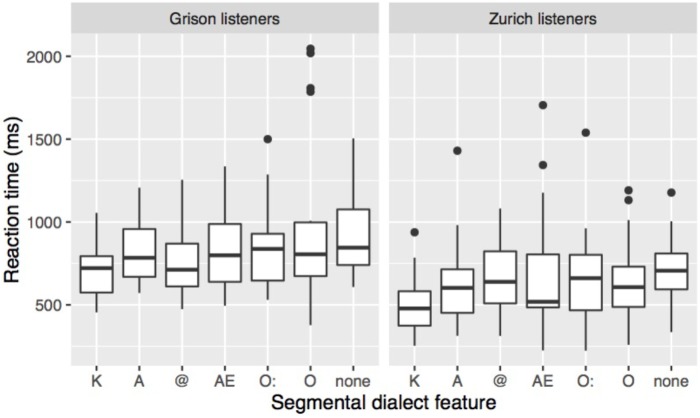
RT in response to the different segmental dialect features in the stimulus. Stimuli with two segmental dialect features have been removed prior to averaging response times over listener and dialect feature.

The effect of the specific dialect feature on RT is shown in **Figure 6**. Both listener groups were fastest in responding to stimuli with K, and slowest in stimuli without segmental dialect feature, while differences among the other categories are not clearly apparent. Linear mixed-effect models were performed on the transformed RT data with listener dialect, dialect feature, and stimulus duration as fixed factors. The maximal converging model with the best fit included random intercepts for speaker, word, and listener. Since there was a significant interaction between a listener’s dialect and dialect feature (χ^2^[6] = 16.3, *p* < 0.05), *post hoc* Tukey tests using the multcomp package in R (Hothorn et al., 2008) were carried out. They confirmed that participants of both dialects responded significantly faster to stimuli containing K than to stimuli without segmental dialect features. Zurich participants further showed significant differences for the contrasts K - @, K - AE, and K - O:, and Grison participants, for the contrast K - O.

In summary, the two listener groups could distinguish more easily and quickly between GRG and ZHG the more segmental dialect features a stimulus contained. Dialect recognition was particularly straightforward in stimuli containing K, A, or @. Similar but less clear-cut patterns arose from the analysis of RTs with shortest response latencies when listeners reacted to stimuli containing K. Although the dialect features affected Grison and Zurich listeners’ answers in a very similar way, there were two major differences between the two listener groups. Overall, Zurich listeners responded faster, and in stimuli without or with one segmental dialect feature, their sensitivity was higher than that of Grison listeners. Based on earlier work (Clopper and Pisoni, 2004a; Díaz Campos and Navarro Galisteo, 2009) we would expect that persons who are more frequently exposed to both dialects have less difficulties distinguishing them. The post-task questionnaire data was used to explore whether differences in exposure are able to explain the different performance of Zurich and Grison listeners. In the post-task questionnaire the participants were asked how often they heard GRG and ZHG in their daily life. The majority of the Grison participants (18/21) reported being exposed to ZHG several times a week, in contrast to the Zurich group from which the majority (19/23) indicated hearing GRG only a couple of times per month or less. Interestingly, despite being more frequently exposed to ZHG than vice versa, Grison listeners were less successful and slower at distinguishing between the two dialects. The between-dialect differences in RT and sensitivity thus do not seem to be an artefact of differences in degree of exposure.

### Role of Acoustic Distance

So far, we only tested the effect of dialect feature on dialect categorization, without considering the actual acoustic properties of the stimuli. The aim of this section is to explore whether the role of the dialect features as found in section “Effect of Segmental Dialect Feature on Dialect Identification” is consistent with the between-dialect acoustic distance in vowel quality. To test whether a more marked vowel quality leads to more correct responses, the Euclidean between-dialect distance in the F1 × F2 vowel space will be used to predict the listeners’ responses. This analysis will be performed across all vocalic dialect features to see whether the contribution of dialect features to dialect identification is consistent with their between-dialect acoustic distance.

**FIGURE 7:**
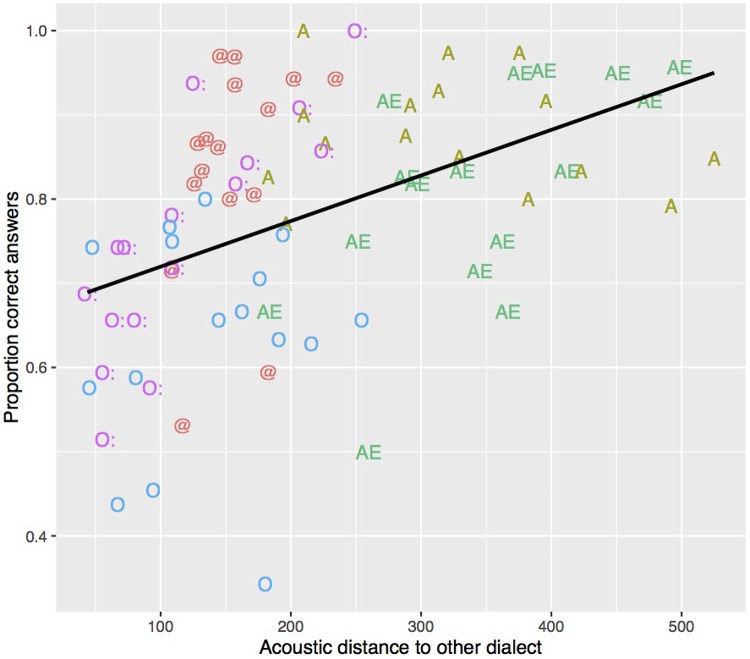
Relationship between the proportion of correct answers (y-axis) and the acoustic distance to the other dialect (x-axis). The proportion of correct answers was calculated separately for each speaker and each dialect feature (i.e., each vowel), resulting in 5 (vowels) × 16 (speakers) = 80 data points. The acoustic distance was measured in mel as Euclidean distance between the target vowel in each stimulus and the centroid for the corresponding vowel in the other dialect. Each value on the x-axis thus represents the averaged Euclidean distance over a vocalic dialect feature and a speaker. For instance, for O, the average distance was calculated for each speaker over the words *Loch*, *Frosch*, and *Schloss*.

**FIGURE 8:**
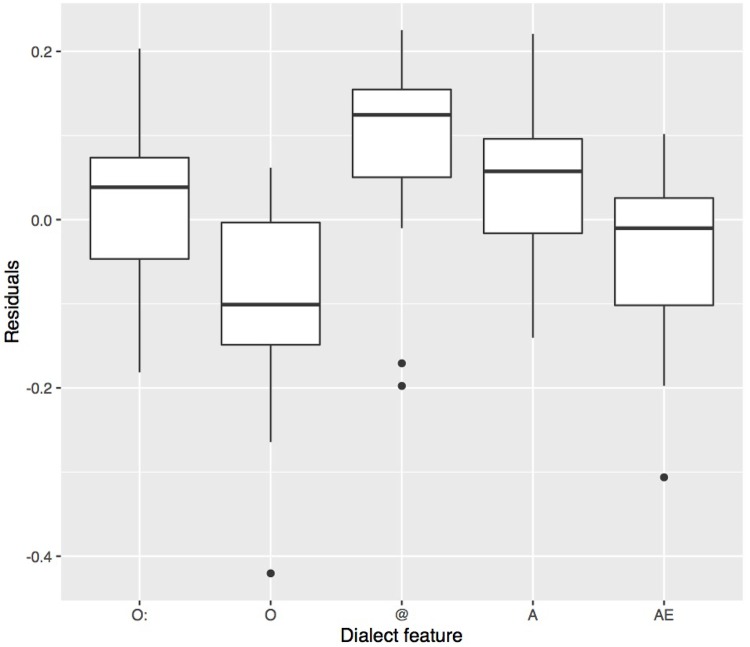
Residuals of the linear model in **Figure 7** predicting the proportion of correct answers from the acoustic distance. Values below zero indicate that, based on the acoustic distance, we would expect more correct answers. Values above zero indicate that, based on the acoustic distance, we would expect less correct answers than were actually observed.

For each stimulus containing one vocalic dialect feature, the acoustic distance between its target vowel and the other dialect’s mean value of the same vowel was calculated (McCloy et al., 2015). This was done by calculating for each target vowel in each stimulus the Euclidean distance to the F1/F2 mean of the same vowel in the other dialect. The first (F1) and the second formant (F2) had been measured over the mid 50% of each vowel using Emu (Harrington, 2010); segment boundaries were set manually. As is apparent from **Figure 7**, stimuli with vowels produced in a more distinct way from the other dialect entailed more correct answers. A, which showed a high d′ value, also displays the greatest between-dialect acoustic distance. A linear model was fitted on the data in **Figure 7** and showed a significant linear relationship between the between-dialect acoustic distance and the proportion of correct dialect answers (*t*[78] = 4.5, *p* < 0.001; *R*^2^ = 0.21). Based on this model, however, we would expect more correct answers for AE and for O than were actually found, and less for @, which shows a rather small between-dialect acoustic distance. One-tailed *t*-tests confirmed that the residuals in **Figure 8** were significantly greater than 0 for @ (*t*[15] = 2.8, *p* < 0.01), and smaller for O (*t*[15] = -3.2, *p* < 0.01), and there was a non-significant trend for A (*t*[15] = 1.6, *p* = 0.06) and AE (*t*[15] = -1.5, *p* = 0.07). Based on Euclidean distance only, O and AE thus should show higher proportions of correct answers, and @ and A should not be recognised as well as they were in the present experiment, indicating that apart from acoustic distance, other factors are at play. The next section will address one potential factor in more detail, namely the role of available knowledge of dialect features.

### Knowledge of Dialect Features

The results in Section “Role of Acoustic Distance” suggested that acoustically measured vowel quality to some extent correlates with accuracy in dialect identification, but at the same time is not sufficient to explain why certain dialect features contribute more to dialect recognition than others. In this section, we will compare the results of the forced-choice experiment with the explicit knowledge speakers have about the two dialects to shed light on the role of top–down processes in dialect recognition.

Swiss German speakers’ knowledge of Grison and Zurich German dialect features was collected in two separate online questionnaires from independent participants who did not participate in the forced-choice categorization task. This procedure was used to avoid priming the listeners on specific dialect features (and possibly affecting their responses in the experiment), and to avoid their explicit answers to be influenced by the experiment itself. Two versions of the same online questionnaire were created, one to collect lay persons’ knowledge of GRG, the other to collect data on the knowledge about ZHG. With the exception of the core question (see below), the questionnaires were exactly the same. They were distributed among native speakers from all over German-speaking Switzerland via contacts of the author. For this part of the study, the external perspective on the dialects is of interest. For this reason, GRG speakers were excluded from the GRG version, and ZHG speakers from the ZHG version of the questionnaire.

The core question of the online questionnaire was: *Welche Merkmale sind typisch für das Bündnerdeutsche [Zürichdeutsche]? Konzentriere dich auf die Aussprache.* ‘Which characteristics are typical of the Grison German [Zurich German] dialect’? Please focus on the pronunciation.’ To facilitate the interpretation of answers, participants were asked to provide an example for each dialect feature. In addition, some basic personal data (age and gender) and information about the participants’ linguistic background (place where they grew up and place of residence) were collected. For the sake of comparability with the experimental data, a first analysis considers only responses from younger GRG and ZHG participants (age range: 18–35). 38 younger speakers of ZHG filled out the questionnaire about GRG, and 34 younger speakers of GRG filled out the version on ZHG. Responses from older participants and people from other dialect regions will be analysed in a second step.

The characteristics mentioned by the participants were grouped into linguistic categories (e.g., *Ch wird als weiches k ausgesprochen* ‘Ch is pronounced as a smooth k’, was translated into K). Cases which were not clearly identifiable with a linguistic category (e.g., *Viele a’s und o’s* ‘many a’s and o’s’) were left unclassified and discarded from the analysis. The most-frequently mentioned features for ZHG were the velar fricative (i.e., K; 23 mentions), the particle/adverb *nöd* (eight mentions), and the *ä*-quality of word-final vowels (i.e., @; seven mentions). The more-lowered /æ/ instead of /ε/ (i.e., AE) was mentioned by three, closed /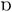/ (i.e., A) by two, and the lack of the split between /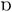:/ and /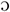:/ (i.e., O:) by one person only. To describe GRG, Zurich participants mentioned *k* (i.e., K; 27 mentions), *a*-quality of word-final schwa (i.e., @; 12 mentions), long vowels (i.e., open syllable lengthening; nine mentions), and the open quality of *a* (i.e., A; four mentions). Other, acoustically quite distinct features of GRG (in comparison to ZHG), namely /ε/ instead of /æ/ (i.e., AE; three mentions) and the split of /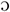:/ and /a:/ (i.e., O:; one mention) were brought up by few participants only. Interestingly, none of the Grison or Zurich participants referred to closed/open /o/ (i.e., O). This is in line with the low d′ value found for stimuli containing O, and might be due to the fact that [o] does occur in GRG as a variant of O (see **Table 1**), and is therefore not exotic to a Grison listener’s ear. The fact that not all dialect-specific features receive the same degree of awareness is not surprising. However, it is interesting to see that the degree of awareness of certain dialect characteristics is able to explain the discrepancy between acoustic distance and dialect recognition described in section “Role of Acoustic Distance.” Most Grison and Zurich people who filled out the online questionnaire were able to mention at least one pronunciation feature, but overall, more features were listed for GRG (median: 2, mean: 2.2) than for ZHG (median: 1, mean: 1.8; one-sided Wilcox rank sum test: *p* < 0.05).

As a second step, the answers from all participants will be analysed. In total, 186 people from all over German-speaking Switzerland filled out the GRG questionnaire, and 209 people filled out the ZHG questionnaire. The number of mentioned dialect features was higher in the GRG than in the ZHG questionnaire (mean_GRG_ = 1.8, mean_ZHG_ = 1.6; one-sided Wilcox rank sum test: *p* < 0.05). This indicates that speakers of Swiss German possess more knowledge about GRG than about ZHG. The number of participants who completed the questionnaire without mentioning any feature was significantly higher in the ZHG (47; 22%) than in the GRG questionnaire (25; 13%), as shown by a Chi-squared test (χ^2^[1] = 4.8, *p* < 0.05). These results are in line with lay persons’ descriptions of ZHG as a neutral dialect (Werlen, 1985; see section “Grison and Zurich German”). Furthermore, participants from all over German-speaking Switzerland agreed more upon the features for GRG, with K and @ being listed by 63 and 35% of the participants, respectively, followed by the diphthongs /i, u/ (9%), and vowel lengthening (9%). The features mentioned for ZHG were more diverse, with K (23%), alveolar /r/ (16%), closed quality of high vowels (14%), and open /æ/ (14%) being the most-frequently mentioned features. The greater agreement upon specific GRG features suggests that there is shared knowledge among Swiss German speakers of how GRG sounds and of what the specific features of this dialect are. ZHG, in contrast, seems to be described by contrasting the dialect with one’s own, and thus naturally leads to a greater diversity of mentioned features.

## Discussion

The aim of the present study was to quantify the contribution of specific segmental cues to dialect recognition, and to investigate the extent to which their effect can be attributed to knowledge about the dialects or to acoustic distance between them. A forced-choice dialect identification task with unmanipulated recordings from 16 speakers was used. The results showed that the specific segment occurring in the stimulus significantly affected how fast and how accurately a speaker’s dialect was identified. Despite the fact that even single words may include prosodic cues to the regional or ethnic origin of a speaker (e.g., Purnell et al., 1999; Atterer and Ladd, 2004), recognition rate and accuracy increased with an increasing number of segmental cues in the stimulus. This finding shows that operationalising the perceptual salience of segmental cues is possible using natural, unmanipulated recordings of isolated words.

Overall, Grison and Zurich listeners relied on the same segmental cues for distinguishing between the two dialects. According to the results, K, realised as (aspirated) stop in GRG and as fricative or affricate in ZHG, appeared to be the perceptually most salient difference between the dialects. Given its acoustic properties and prominent position syllable- or word-initially, this finding is in line with what would be expected by auditory principles: an abrupt transition between the closure phase and release of the plosive, combined with a prominent position, favour the perceptual salience of that sound (Ohala and Kawasaki, 1984; Bladon, 1986; Auer, 2014). On the contrary, the contribution of the different vocalic cues in dialect identification was only partly consistent with what would be predicted by acoustic principles. The important role of A is not surprising given the marked acoustic distance between GRG [a] and ZHG [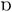]. However, based on acoustic distance alone, O should contribute more, and @ less to dialect identification than they actually did in the current experiment.

An online questionnaire with unrelated participants showed that the discrepancy between acoustic distance and perceptual salience can be explained in terms of differences in knowledge of GRG and ZHG dialect features. Participants from Zurich most frequently mentioned K, A, and word-final @ when asked about typical GRG features, whereas none of them mentioned O. The interaction between dialect feature and acoustic distance suggests that phonetic distance is weighted more when occurring in a sociolinguistically salient feature (i.e., in @), and less so when occurring in a phoneme for which listeners are not aware of dialectal variation (i.e., O). Listeners thus seemed to follow their prior knowledge where to expect variation, and weighted acoustic distance differently depending on whether or not dialect variation for a certain phoneme is expected.

At this point the question arises why K and @, but not O: or O, acquired sociolinguistic salience in the first place. According to Auer (2014), three main aspects contribute to a linguistic feature’s salience: acoustic-auditory factors, cognitive factors, and sociolinguistic factors. Given the unstressed nature of @, acoustic-auditory factors are certainly of less importance here. Instead, we argue that cognitive factors are at work. While ZHG shares several phonetic features with neighbouring dialects, GRG is spoken in a small area and shows less phonetic similarity with other dialects, as illustrated by dialectrometric similarity maps (Scherrer, 2007).^1^ In particular, with word-final /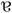/ (@) and word-initial /k^h^/ (K) GRG possesses two locally very restricted phonological forms that do not occur in other dialects. We suggest that this may be the reason why people from all over German-speaking Switzerland described GRG in a very similar way, and why this dialect is generally perceived as a marked, rather than a neutral dialect. Jaeger and Weatherholtz (2016) argue that, in an initial phase, linguistic variants the listener has less experience with are less expected and therefore lead to greater surprisal – a precondition for the variant to acquire sociolinguistic salience at later stages. The locally restricted forms of GRG in an initial phase thus attract Swiss German speakers’ attention because, given their restricted areal distribution, they are unexpected. Following Jaeger and Weatherholtz’s (2016) argumentation, the high frequency of occurrence of K and @ in later stages would facilitate that listeners “learn” and associate these variants with the dialect, and allow them to acquire sociolinguistic salience.

The experiment also provided more general insights into the process of dialect identification. At first sight, listeners appeared to better identify their own as opposed to the other dialect, as reported in several dialect recognition studies on other languages (Williams et al., 1999; Gooskens, 2005; Boomershine, 2006; Baker et al., 2009; Yan, 2015; Avanzi and Boula de Mareüil, 2017). Our results and, in particular, the use of signal detection theory, offer an alternative interpretation. Rather than more easily *recognising* their own dialect, listeners in the present study were *biased* towards ascribing an ambiguous stimulus to their own dialect. Therefore, the results invite reconsideration of the findings of the earlier research mentioned above. In fact, an own-group bias has also been demonstrated when participants were asked to estimate speaker age from voices (Moyse et al., 2014), and seems to be a more general component of speech processing and social categorization. Recently, Bestelmeyer et al. (2015) found that participants showed an enhanced neural response when listening to their own accent, but not when listening to other regional varieties of English. With their study, the authors provided neural evidence for the general observation that in-group accents are preferred over out-group accents. Perrachione et al. (2010) observed that listeners were better at remembering voices that were perceptually similar to their own ethnic accent. However, there is also some evidence for variation in the own-dialect response bias. Avanzi and Boula de Mareüil (2017), who compared the effect of speaker and listener dialect on accent identification in French, found that the tendency to better identify one’s own as opposed to other dialects was more marked in Swiss than in Belgian and French participants. Although this interpretation remains speculative, it is possible that the federal, non-centralistic political organisation of Switzerland, together with the lack of an oral standard variety with high prestige, further enhances this own-dialect bias. Further research is needed to understand the relationship between own-dialect biases, social categorization, and speech processing.

Apart from a general own-dialect response bias, the use of signal detection theory revealed an effect of a listener’s dialect on their response bias, the latter being less marked in Grison than in Zurich participants. Given the much larger population of the Zurich area in comparison to the Chur Rhine valley, people from the latter region might simply be more likely to meet someone from Zurich than vice versa (see Perrachione et al., 2010, for a similar argument why Americans are more likely to ascribe an amgibuous linguistic stimulus to a Caucasian than an African American speaker). As reported in section “Effect of Segmental Dialect Feature on Dialect Identification,” such a difference in degree of exposure to the two dialects indeed exists between our listener groups. Zurich and ZHG thus may be more present and activated for Grison participants than the Grisons and GRG for persons from Zurich. Another possible explanation for the less marked own-dialect bias in Grison listeners, once again, is the different values ascribed to the two dialects. Taken together, the descriptions for ZHG listed by Werlen (1985) indicate that ZHG is considered to be a neutral, and GRG a marked dialect. Further evidence for this classification comes from our online questionnaires, in which participants from all over German-speaking Switzerland mostly agreed on “typical GRG” features and seemed to possess shared knowledge about this dialect. In contrast, the features mentioned for ZHG largely depended on a participant’s dialectal background, suggesting that ZHG is described mainly by contrasting the dialect with one’s own. This interpretation is further supported by work showing that among eight Swiss German dialects, ZHG was recognised the worst (Guntern, 2011). Thus, although ZHG has neither overt prestige nor the status of a standard language, its conception of a “neutral dialect” possibly explains why Grison participants chose their own dialect not as often as Zurich participants. In line with this interpretation, Yan (2015) found a response bias for the more neutral and prestigious Enshi variety in her dialect categorization task on Enshi Mandarin dialects, and Avanzi and Boula de Mareüil (2017) observed that French listeners were biased towards selecting the Paris accent, which is the closest to the French standard. To better differentiate between an own-dialect bias and a bias towards a more neutral or more prestigious linguistic variety, future experiments ideally would include a third listener group, for instance, from Berne or Basel. Additionally, the choice between “Dialect x” and “Not dialect x” could be used (see Llamas et al., 2016) instead of the dialect labels.

The two listener groups differed not only in the magnitude of their response bias, but also in RT and sensitivity. Unexpectedly, and despite a more pronounced own-dialect bias, Zurich participants overall responded faster and were more sensitive to the dialect differences than Grison participants. Unlike in earlier work (Clopper and Pisoni, 2004a; Díaz Campos and Navarro Galisteo, 2009), a different amount of exposure to the dialects was not able to explain the differences in the current experiment. One possible reason for this result is that, given the specific situation of German-speaking Switzerland (see section “Sociolinguistic Situation of German-Speaking Switzerland”), most speakers already possess at least some amount of knowledge about how different dialects sound. We do not currently have an explanation for the higher sensitivity of Zurich listeners in the dialect identification task. An interesting working hypothesis for future research would be that speakers in urban areas, who are in contact with many different dialects and accents on a daily basis, might be more sensitive to linguistic variability *per se*.

At least three explanations seem conceivable to explain the faster response times of Zurich listeners. First, they might result from the higher own-dialect response bias of this group. Given that RT was calculated on correct responses only, its higher amount of correct responses to ZHG stimuli would have led to a generally faster response latency. However, since Zurich listeners were faster not only in the ambiguous, but across all three conditions, this interpretation does not seem plausible. A second, but arguably speculative explanation is that the between-group differences in RT result from cultural differences, given that articulation rate for the Zurich area was found to be faster as well (Leemann, 2016; see Ebersbach et al., 2000 for differences between people from Berlin and Tyrol in gait velocity). A third and more plausible explanation for the difference in response latency may be different decision strategies. If Zurich listeners identified the more marked Grison dialect directly – without comparing it with their own dialect – they should display faster response times than Grison listeners, who possibly identified ZHG by first contrasting the stimulus against their own dialect, which arguably is more time-consuming.

## Conclusion

The experiment showed that native speakers of Grison and Zurich German can quite accurately distinguish between these two dialects based on isolated words which differ from their own dialect in one or two segments. In words without a segmental dialect difference, listeners were basically at chance level, confirming earlier work showing that for dialect identification, segmental cues are key (e.g., Van Bezooijen and Gooskens, 1999; Gooskens, 2005; Fuchs, 2015; Leemann et al., 2016). The method used in this study provides a way to operationalise and quantify perceptual salience of segmental cues with natural, unmanipulated stimuli. According to the results, /k^h^, kk/-/x, kx/, /a/-/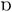/, and syllable-final /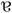/-/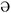/ are the most salient differences between the two dialects. This result reflects Swiss Germans’ explicit knowledge and expectations about how Grison and Zurich German sound, and supports earlier results that a few segmental properties are sufficient to recognise a familiar linguistic variety (Clopper and Pisoni, 2004b). As expected, a greater acoustic distance between the dialects facilitated their distinction. This correlation was more marked in dialect differences listeners are aware of. Unlike most previous research on dialect identification, this study relied on signal detection theory for data analysis. The method proved to be very useful in analysing differences between the listener groups, and to detect a marked own-dialect response bias in almost all participants. This response bias suggests that, rather than more easily recognising their own dialect (as suggested by Williams et al., 1999; Baker et al., 2009; Avanzi and Boula de Mareüil, 2017), listeners are biased towards ascribing an ambiguous stimulus to their own dialect.

## Ethics Statement

This study was carried out in accordance with the recommendations of the Ethics Committee of the Faculty of Arts and Social Sciences at University of Zurich with written informed consent from all subjects.

## Author Contributions

HR designed and conducted the research, analysed the data, interpreted the result, and wrote and edited the paper.

## Conflict of Interest Statement

The author declares that the research was conducted in the absence of any commercial or financial relationships that could be construed as a potential conflict of interest.

## Footnotes

1 The maps are based on data from the Atlas of German-speaking Switzerland (Hotzenköcherle et al., 1962) and were generated using all phonological variables.
